# A Novel Minimally Invasive Technique to Create a Rabbit VX2 Lung Tumor Model for Nano-Sized Image Contrast and Interventional Studies

**DOI:** 10.1371/journal.pone.0067355

**Published:** 2013-06-28

**Authors:** Takashi Anayama, Takahiro Nakajima, Michael Dunne, Jinzi Zheng, Christine Allen, Brandon Driscoll, Douglass Vines, Shaf Keshavjee, David Jaffray, Kazuhiro Yasufuku

**Affiliations:** 1 Division of Thoracic Surgery, Department of Surgery, Toronto General Hospital, University of Toronto, University Health Network, Toronto, Canada; 2 Department of Radiation Physics, Radiation Medicine Program, Princess Margaret Hospital, University Health Network, Toronto, Canada; 3 Leslie Dan Faculty of Pharmacy, University of Toronto, Toronto, Canada; Northwestern University Feinberg School of Medicine, United States of America

## Abstract

**Background:**

The rabbit VX2 lung cancer model is a large animal model useful for preclinical lung cancer imaging and interventional studies. However, previously reported models had issues in terms of invasiveness of tumor inoculation, control of tumor aggressiveness and incidence of complications.

**Purpose:**

We aimed to develop a minimally invasive rabbit VX2 lung cancer model suitable for imaging and transbronchial interventional studies.

**Methods:**

New Zealand white rabbits and VX2 tumors were used in the study. An ultra-thin bronchoscope was inserted through a miniature laryngeal mask airway into the bronchus. Different numbers of VX2 tumor cells were selectively inoculated into the lung parenchyma or subcarinal mediastinum to create a uniform tumor with low incidence of complications. The model was characterized by CT, FDG-PET, and endobronchial ultrasound (EBUS). Liposomal dual-modality contrast agent was used to evaluate liposome drug delivery system in this model.

**Results:**

Both peripheral and mediastinal lung tumor models were created. The tumor making success rate was 75.8% (25/33) in the peripheral lung tumor model and 60% (3/5) in the mediastinal tumor model. The group of 1.0×10^6^ of VX2 tumor cells inoculation showed a linear growth curve with less incidence of complications. Radial probe EBUS visualized the internal structure of the tumor and the size measurement correlated well with CT measurements (r^2^ = 0.98). Over 7 days of continuous enhancement of the lung tumor by liposomal contrast in the lung tumor was confirmed both CT and fluorescence imaging.

**Conclusion:**

Our minimally invasive bronchoscopic rabbit VX2 lung cancer model is an ideal platform for lung cancer imaging and preclinical bronchoscopic interventional studies.

## Introduction

Lung cancer is the leading cause of cancer-related death in the Western world accounting for 28% of all cancer deaths. Recent clinical trials suggest that annual low dose x-ray computed tomography (CT) screening increases the incidence of lung cancer cases while decreasing the number of mortality [1]. Early diagnosis of lung cancer can lead to more treatment options, less invasive surgery, and a higher survival rate. Further advancement in lung cancer imaging may improve the outcomes and therapies.

Recently there has been a growing interest in the employment of nanotechnology-based agents for longitudinal imaging applications. These nano-sized agents have the ability to accumulate and be retained at malignant tumor sites through the enhanced permeability and retention effect [2], [3]. For example, liposome-based contrast agents have shown to successfully accumulate in tumors and provide prolonged signal enhancement of target lesions [4]. These are most suitable for applications which require longitudinal and repeated imaging such as image-guided surgical interventions. However, with respect to lung cancer, previous investigations have been primarily performed in rodent models [5]–[7]. For successful clinical translation and validation of new imaging and endobronchial intervention tools, a reproducible lung cancer model in a larger animal is required. This type of model should provide a comparable setting to that of a human in order to allow for evaluation of the changes in both the objective tumor and the surrounding tissues and organs pre, during and post-treatment.

VX2 is a rabbit squamous cell carcinoma which has been used as a model for malignancies in several organs including the liver, muscle, rectum, kidney, and lung [8]–[12]. The VX2 lung cancer is a relatively large animal model for relevant preclinical imaging and interventional studies for lung cancer [13]–[16]. However, previously reported transthoracic VX2 inoculation techniques require a mini-thoracotomy which is associated with unfavorable accumulation of the imaging agent to the wound site and it also carries risk of pneumothorax and pleural dissemination [14]. The fluoroscopy-guided transbronchial catheter VX2 implantation technique required percutaneous cannulation [17]. Controlling the size of the VX2 tumor is a common issue as the fast growing nodules affect the comparative imaging studies and may cause complications for survival studies due to the aggressive nature of the VX2 tumor.

The purpose of this research is to employ a minimally invasive technique to develop a reproducible lung cancer animal model in rabbits suitable for imaging and transbronchial interventional studies using clinically relevant tools. The number of inoculating tumor cells was optimized to create a uniform lung tumor with decreased incidence of complications. In addition, the radiological features of the lung VX2 tumor were described using a nano-sized liposome-based contrast agent in CT and fluorescence optical imaging.

## Materials and Methods

### VX2 Cancer Cell Preparation

Frozen VX2 cancer was extracted from frozen archives which had been used in previous research [4], [18]. Frozen VX2 tumor tissue kept in −80 degree Celsius was thawed, minced, and isolated with a 100 µm cell strainer to obtain a uniform single-cell suspension from tissues. 1.0 ×10^7^ of cells suspended in 500 µl of HBSS solution was injected into donor animals’ right lateral quadriceps. Three weeks following inoculation, the intramuscular tumor reached approximately 30 mm was then isolated again, then suspended in extracellular matrix protein (Matrigel™, BD, Mississauga, Ontario, Canada) at different concentrations described later.

### Bronchoscopic VX2 Innoculation

**Figure 1:**
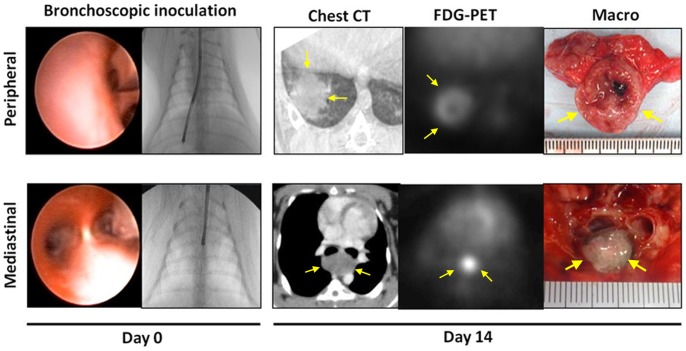
Ultra-minimally invasive syngeneic VX2 lung cancer model in rabbits. VX2 squamous cell carcinoma cells were inoculated into either the lung parenchyma or mediastinum with an ultra-thin bronchoscope and a dedicated needle. Follow-up CT showed the tumor growing in either the lung parenchyma or the mediastinum. By FDG-PET, each lung tumor was visualized as the solitary hot spot. Macroscopic axial cross section of the tumor showed the solid VX2 tumor.

New Zealand white rabbits were employed in the study to make the animal model bearing each solitary lung tumor. The animals (2.5–2.8 kg) received ketamine (50 mg/kg) and xylazine (5 mg/kg) followed by induction of anesthesia with 5% isoflurane. A miniature laryngeal mask was inserted for ventilation and bronchoscopic access. General anesthesia was maintained with 2% isoflurane and 2 L/min oxygen. A 21 gauge bronchoscopic needle (Olympus MAJ-65) was integrated into the accessory channel of the ultrathin bronchoscope (Olympus BF-XP160F) with a retrograde integration technique. The bronchoscope was led to the right peripheral bronchus. The bronchoscopic needle was exposed 5 mm from the tip of bronchoscope, then the bronchial wall was penetrated by the needle (figure 1). VX2 tumor cells/Matrigel suspension was inoculated into the peripheral lung parenchyma with the volume of 200 µL. After inoculation, the laryngeal mask was removed and the animals were given 100% oxygen and monitored using a pulse-oximeter until they demonstrated complete recovery from anesthesia. The complications of the procedure were accessed immediately after the procedure. The endobronchial bleeding was accessed bronchoscopically. No occurrence of pneumothorax was confirmed by auscultation. The overall respiratory function was accessed by non-invasive pulse oximetry at ear auricle artery for more than 30 minutes.

### The optimization of Lung Tumor Models

**Figure 2:**
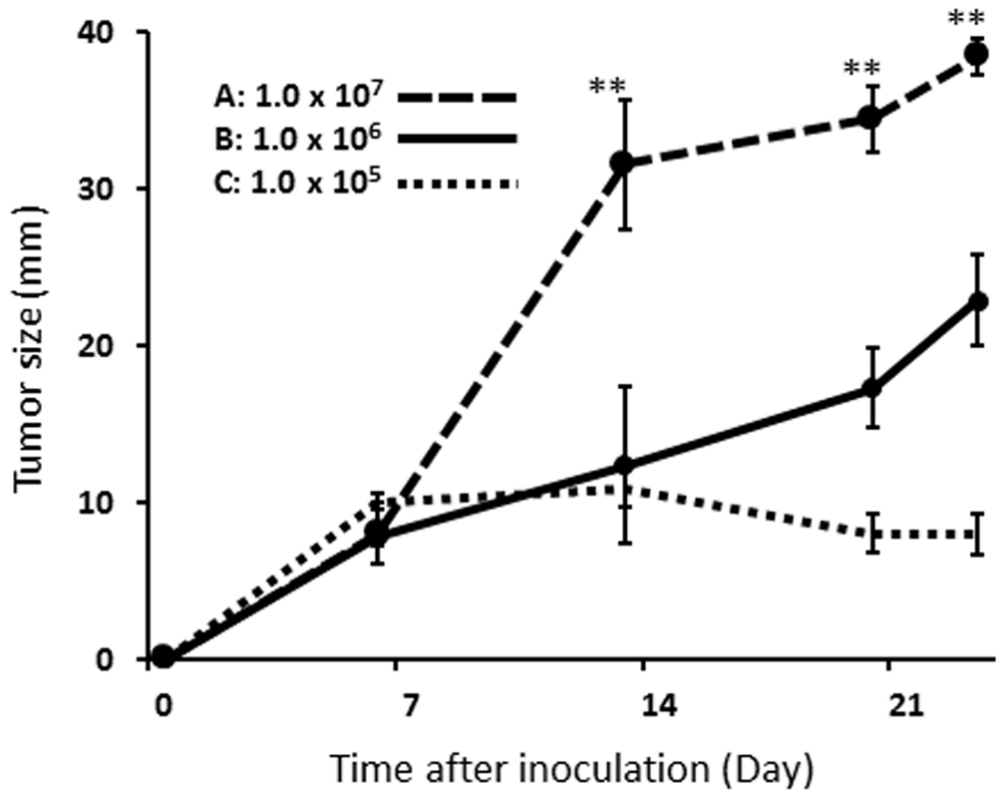
Growth curve of each set of rabbits with a VX2 tumor. Different number of VX2 tumor cells was inoculated into the lung. Group A grew more than 30 mm in diameter during the first 14 days after tumor inoculation, followed by the major complications. Group B showed linear growth curve with less incidence of complications during the study period. Group C showed low tumor making success rate. Tumor size was measured by CT using the maximal axial slice. The values for each group in the figure stand for the averages of all animals with peripheral lung tumor in each group (n = 25 in total. Group A: n = 4, Group B: n = 19, Group C: n = 2). Error bars represent one standard deviation of uncertainty. On Day 14 and later, the tumor size of group A was significantly larger than that of other groups (**p<0.01, Mann Whitney U test).

Three different amounts of tumor cells (group A: 1.0×10^7^, group B: 1.0×10^6^, group C: 1.0×10^5^) were inoculated to the peripheral lung parenchyma with a volume of 200 µL of Matrigel. Considering the reported tumor making success rate of between 46–80% [13], [14], [16], we used 4 rabbits for each group in order to expect more than 3 successful lung tumor models. The state of lung tumors were checked weeky by CT (figure 2). Reflecting the poor tumor development in group C, 2 animals were added to group C. When making the subcarinal mediastinal lung cancer model, the same tumor cell density as group B was inoculated to the subcarinal mediastinum of 5 animals.

### Radial Probe EBUS Examination

**Figure 3:**
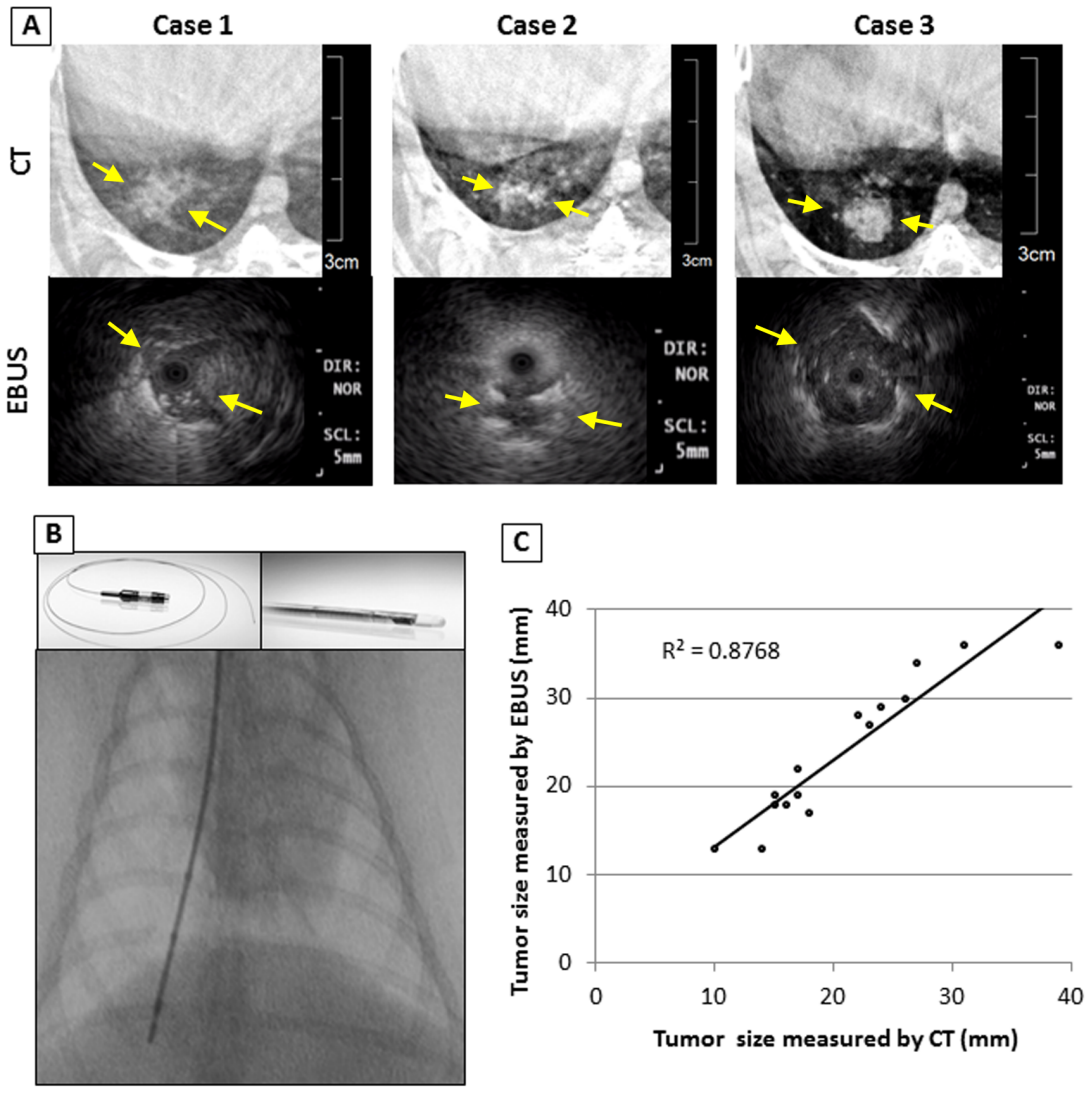
Comparison of CT and EBUS images. **A:** CT and EBUS images of the 3 representative cases. EBUS visualized axial images of the lung tumor. The tumor was characterized as the iso-echoic heterogeneous mass lesion with high echoic margin. **B:** EBUS was led to the lung tumor under fluoroscopy guidance. **C:** EBUS measurement of tumor size was strongly correlated with that of the CT ((n = 16, R^2^ = 0.8768; R^2^: the Pearson Coefficient of Determination).

The radial probe endobronchial ultrasound (EBUS) of 2.6 mm in diameter (UM-S20-20R, Olympus, Tokyo, Japan) was inserted into the bronchus through the airway and was directed to the peripheral bronchus adjacent to the lung tumor under fluoroscopic guidance (Figure 3). Ultrasound images were obtained with an ultrasound image processor (EU-ME1, Olympus, Tokyo, Japan).

### Liposome Contrast CT and FDG-PET Studies

**Figure 4:**
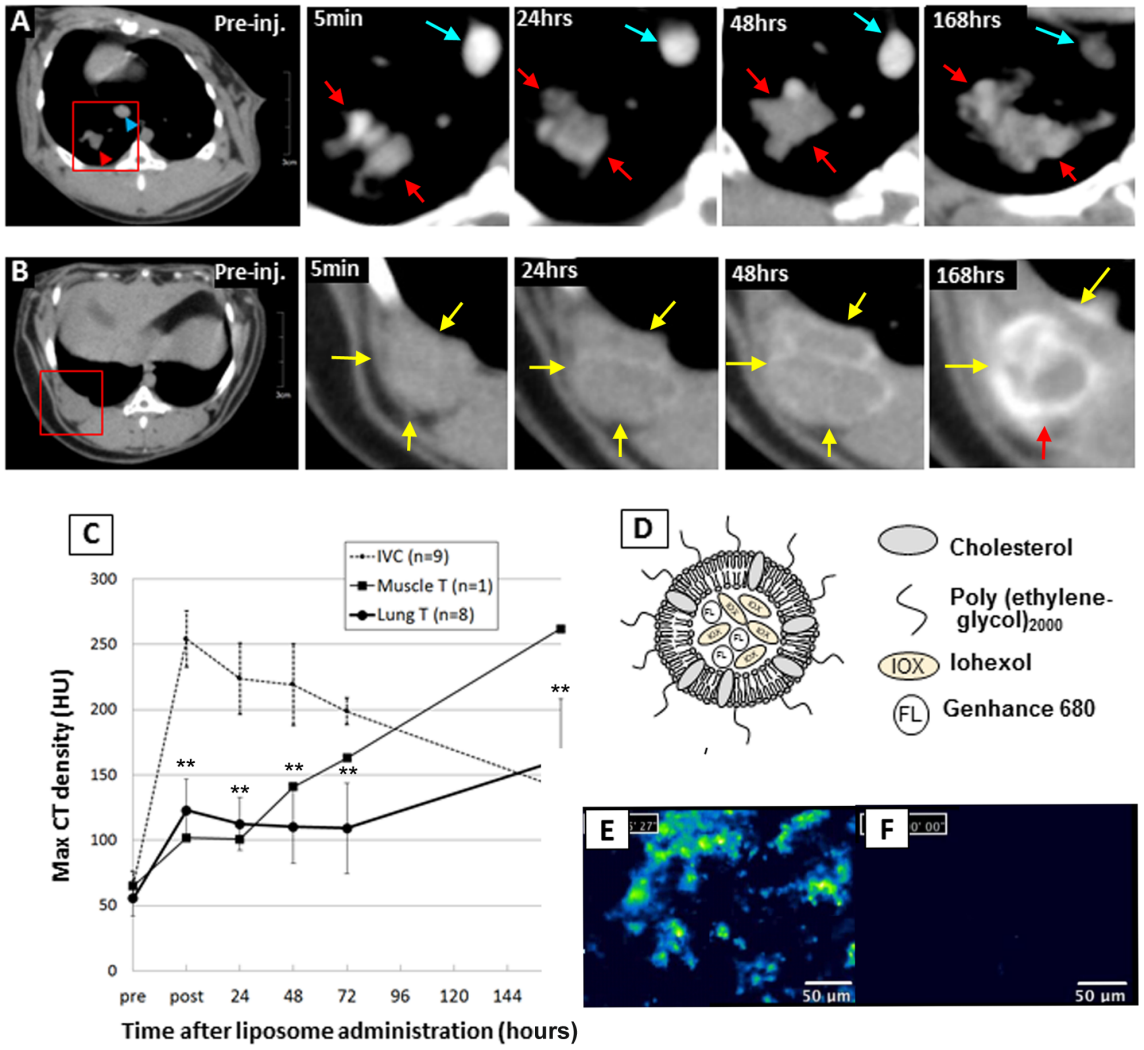
CT contrast enhancement kinetics and fluorescence signal of the CT/fluorescent liposome accumulation in VX2 lung tumor. A: Chronological enhancement effect of the systemically administrated liposomal dual modality contrast for VX2 lung tumors evaluated by periodic CT scans. Iodine concentration in the specified area of the tumor (red arrows) and inferior vena cava (blue arrows) was determined by first defining a region of interest (ROI) and then measuring the Hounsfield unit (HU). B: The one case of muscle VX2 tumor case was shown as a reference (yellow arrows). C: The VX2 lung tumors were kept enhanced at a level of more than 100 HU and maintained at the same level until they showed the highest contrast effect at 168 hours after administration. The marginal part of a muscle VX2 tumor tended to be enhanced higher than lung tumors. IVC showed the highest peak right after administration, followed by decrease over time. D: Schematic drawing of the dual modality liposome agent co-encapsulating iohexol (CT agent) and Genhance 680® (fluorescence agent). E: Fluorescence micro-endoscopy detected the accumulation of liposomes in the lung tumor nodule in the excised lung tissue. F: On the other hand, the normal lung excised from the same animal exhibited considerably few signals of liposomes.

Chest micro-CT scans were acquired weekly throughout the course of the experiment using the GE Locus Ultra scanner (GE Healthcare, Milwaukee, WI, USA) operated at 80 kVp and 50 mA. Each rabbit was anesthetized with a mixture of 2% of isoflurane and 2 L/min of oxygen via a nose cone by inhalation during each imaging session. For the FDG-PET scans, animals were fasted for 12 h prior to imaging and their blood glucose level was measured immediately before FDG administration using a blood glucose meter (Ascensia Contour®, Bayer Healthcare LLC, Mishawaka, IN, USA). The mean blood glucose level for the five rabbits was within the normal range (5.2±0.6 mmol/L). Each animal received 30 MBq of ^18^F-FDG as an intravenous administration via the marginal ear vein. FDG-PET scans were acquired 60 min post FDG administration using the clinical GE Discovery ST PET/CT scanner (General Electric Medical systems, Milwaukee, WI, USA). The dual-modality CT/optical liposome agent (Figure 4) was manufactured using the same protocol as previously reported [19], [20]. Specifically, the liposome agent (86±3 nm in diameter) contained 50 mg/mL of iodine in the form of iohexol (Omnipaque, GE Healthcare, Milwaukee, WI, USA) and 6.0±1.0 µg/mL of Genhance680® (Perkin Elmer, USA). Twenty-five to 27 mL of the CT/optical liposome agent was administered as a slow bolus injection (0.5 mL/s) to each rabbit via the marginal ear vein catheter. CT imaging (Aquilion One, Toshiba, operated at 120 kVp, 200 mA) was performed daily for 7 days (168 hours) post-liposome administration. At the study endpoint, following euthanasia, fluorescence detection of the liposome agent accumulation in the lung tumor was performed using a laser fiber confocal microscopy system (Leica FCM1000 with the ProFlex S-0650 650 µm fiber optic probe).

### Experiment Group and Animal Number

In total, 38 animals were used for this study. 25/33 animals developed peripheral lung tumors and 3/5 animals developed mediastinal tumors, while 10 animals did not develop any tumors. Among 25 of the peripheral lung tumor models, the first 10 were used for the optimization of peripheral lung tumor models, 3 were used for the FDG-PET study, 4 were used for the EBUS study, and 8 were used for the liposome study. Among 3 of the mediastinal lung tumor models, 1 was used for the FDG-PET study.

For the liposome study, power analysis was used to determine an appropriate number of animals to use in the liposome contrast study in order to detect a biologically important effect. In the first 3 cases, the HU values of the peripheral lung tumors were 54±16.8 before liposome contrast administration and 107.7±10.1 at a point of 24 hours after liposome administration (n = 3). According to these sample data, the estimated effect size was calculated to determine the total sample size necessary to make more than a difference in HU of 30 (In the standard mediastinal window of CT (window width, 400 HU; window center, 30 HU), a difference in HU of 30 or more would be of clinical importance [21]). The sample sized was computed by priori analysis [22] of two sided T test by G*power version 3.1 [23], [24] with the following statistical setting (two tails, α error: 0.05, 1-β error: 0.95). As the result, effect size d was computed as 3.16, then 8 animals were required to maintain the same range of power. Therefore, 8 animals were used in total for the liposome contrast CT study.

### Statistical Analyses

The Mann–Whitney U test was used to compare continuous variables such as the tumor size (figure 2) and Hounsfield unit (HU) value of CT (figure 4C). The square of the Pearson correlation coefficient (R^2^) was computed in figure 4 between the observed and predicted data values. Statistical analysis was performed using the Statistical Package for Social Sciences, version 15, statistical software for Windows (SPSS, Chicago, Ill).

### Humane Animal Care

The study was conducted under the approval of the animal care committee at the University Health Network. Humane care was provided throughout all animal experiments according to the 1996 Guide for the Care and Use of Laboratory Animals recommended by the U.S. National Institutes of Health.

## Results

### Peripheral Lung Cancer Model

Three different cell densities were inoculated into the peripheral rabbit lung of three animal groups: group A (1×10^7^ cells), group B (1×10^6^ cells), and group C (1×10^5^ cells). In each successful case, the presence of the tumor as a solitary pulmonary nodule was confirmed by CT 14 days post-inoculation (Figure 1; upper row). In the initial study set, the success rates for tumor development was 100% (4/4) in group A, 100% (4/4) in group B, and 50% (2/4) in group C. Group A tumors showed exponential growth curve during the first 2 weeks. The size of group A tumors were significantly larger than group B on day 14 (A: 31.5±4.1 vs B: 12.4±1.49 (mm), each n = 4, p<0.0030) and onwards. Furthermore, two out of 4 cases in group A demonstrated extensive spread of the disease (i.e. massive pleural effusion and pulmonary metastases) by day 14, whereas Group B maintained a high success rate and the tumors showed the arithmetical progression. Group C showed poor tumor growth and unsuccessful tumor development (7.0±4.24 (mm), n = 2). An additional 2 animals were entered in group C, but they also showed unsuccessful tumor development. Therefore cell density B (1×10^6^ cells) was selected as the optimal tumor inoculation protocol for all subsequent studies. The final success rates of the peripheral lung tumor development was 100% (4/4), 82.6% (19/23) and 33.3% (2/6) for groups A, B and C, respectively. The growth curves of each group of tumors were shown in figure 2. On day 14 and later time points, group A tumors were consistently larger than that of group B or group C (*p*<0.01). The tumor doubling times were 1.2 days, 3.6 days and 18.8 days for groups A, B and C during the first 2 weeks after tumor inoculation [25], [26].

### Mediastinal Tumor Model

Based on the results of the peripheral lung tumor model, 1.0×10^6^ of VX2 tumor cells were inoculated into the subcarinal mediastinum using the same bronchoscopic technique. The model making success rate was 60% (3/5, 15.3±7.6 (mm)). Each mediastinal tumor was identified as a subcarinal mass lesion which oppresses the membranous part of bronchus by CT scan on day 14 (Figure 1; lower row).

### FDG-PET Scan

Four animals (3 peripheral lung tumor models were and 1 mediastinal tumor model) were used for the FDG-PET study. Both peripheral lung tumors and a mediastinal tumor exhibited strong FDG accumulation (Figure 1-Day14). The maximum standard uptake value (SUV_max_) for the peripheral and mediastinal tumors were 12.8±4.3 (n = 3) and 10.2 (n = 1), respectively.

### Bronchoscope and Radial Probe EBUS

Bronchoscope and EBUS were performed in the 4 animals in group A in the study of the optimization of lung tumor models, and 4 other animals in group B were used specially for this study (n = 8 in total). Developing solitary peripheral lung tumors were examined by the EBUS study twice on day 14 and 21 after VX2 tumor inoculation. Therefore EBUS was performed for a cumulative 16 times. Significant changes were observed in the peripheral bronchus adjacent to the tumor including deformation, narrowing, white discoloration of mucosa, and dullness of bronchial bifurcation, all of which were caused by the submucosal invasion and progression of the tumor. EBUS allowed for detailed visualization of the morphology of the tumor with distinct margins (Figure 3-A). The internal structure of the tumor was also visualized in details with EBUS. Tumor size measurement by EBUS correlated with CT measurement (Figure 3-C).

### CT and Fluorescence Imaging Using the Dual Modality Liposome Agent

The dual-modality CT/fluorescence liposome agent was intravenously injected, the time dependent liposome contrast accumulation kinetics was followed up with daily CT scans for 7 consecutive days (Figure 4 A-C). The lung tumor nodules accumulated significant amount of the liposome agent achieving significant contrast enhancement in CT (increase up to 67.4±10.1 HU; Hounsfield unit) compared to pre-contrast administration over the 7-day period (*p*<0.0019). The tumor signal enhancement was measured to be the highest on day 7 after administration, while the vascular signal enhancement (measured at inferior vena cava) showed the highest peak immediately after administration following the slowly decrease of enhancement down to day 7. The confocal optical micro-endoscope successfully detected the fluorescence signals from the liposomes accumulated in the tumor tissue (Figure 4E) while almost no fluorescence signal was observed in the surrounding healthy lung and bronchus tissue.

### Complications

The purpose of this research was to create a preclinical lung tumor model which closely mimics that of human lung cancer with the potential for widespread adoption for various imaging studies. Major complications such as fluid collection in the thoracic cavity, chest wall invasion, and existence of pulmonary metastases around the primary tumor would result in ambiguous image findings of the tumor which is not suitable for morphological evaluation in imaging investigations. All cases with complications encountered during the present study were part of animals from Group A. This group of animals had tumors which exhibited exponential growth in the first 2 weeks, and then reached and invaded to the visceral pleura.

## Discussion

This is the first report on the use of bronchoscopic techniques to create solitary lung tumor nodules in a rabbit model which allowed for repeated bronchoscopic/EBUS sessions to be performed as follow-up. The minimally invasive airway management using a miniature laryngeal mask and ultra-thin bronchoscopic VX2 inoculation demonstrated significant advantages compared to previously reported methods in terms of the less invasiveness to the airway and the minimized incidence of complications such as pleural invasion, pleural effusion, and pneumothorax which may adversely affect comparative imaging studies. Under bronchoscopic monitoring, lung tumor cells were securely inoculated at any desired anatomical site in the lung with no chance of intrabronchial dissemination of tumor cells.

The technique also produced the first solitary mediastinal tumor model which could be accessible by bronchoscope. This model mimics the middle mediastinal tumors or bulky mediastinal lymph node metastasis which results in secondary pathological effect such as bronchial stenosis because of the mass effect. The model can be employed for a variety of interventional studies such as endobronchial stenting and endobronchial energy ablation, and the post-therapy changes in both the lung tumor and the surrounding normal tissue can be examined using a bronchoscope and EBUS periodically.

In conjunction with CT, which allows for spatially accurate localization of the tumor nodule with respect to gross anatomy, radial probe EBUS offers visualization of exquisite coaxial morphological details of the tumor including the size, shape, and the fine internal structures.

Through radiological examination, our rabbit VX2 lung tumor model has been extensively characterized. Specifically, it was a FDG positive, vascularised and relatively permeable tumor as it allows for accumulation of the macromolecular liposome-based agent. In order to enhance the ability of non-invasive imaging techniques (i.e. CT and fluorescence-based endoscopy) to monitor disease progression in the lung, a newly developed liposome-based CT/fluorescence imaging agent was administered to the animals. Signal enhancement was as high as 67.4 HU increase in the tumor in CT over the course of 7 days following a single administration of the liposome agent. This allowed for effective non-invasive visualization of the lung tumor nodule over time. Fluorescence endoscopy confirmed the specificity of tumor accumulation of the liposomes in the malignant lung nodule compared to surrounding healthy lung tissue.

Thorough characterization of this liposome agent will contribute to a deeper understanding of the tumor physiology and ultimately enable non-invasive monitoring of the tumor in response to different therapies as well as characterization of tumor margins post-surgery. Results from the present study successfully demonstrated the suitability of employing this newly developed multimodality agent for image-guided intervention applications as it allowed for repeated pre-operative CT acquisition and intra-operative fluorescence-endoscopy imaging with one single administration.

One limitation of this model is that the VX2 tumor is not a human-derived allograft but a rabbit origin neoplasm. As a result, there is little information on the cross reactivity of most commercially available antibody products to VX2 cells, which can limit the use of this model targeted therapeutics which need appropriate antigen-antibody reactions. However, this syngeneic rabbit lung tumor model represents a useful and complementary larger animal model to existing human lung cancer xenografts developed in immune-compromised mice, both in terms of suitability for use in imaging and interventional procedures using clinically relevant systems and for evaluation of host immunity changes during cancer progression and response to therapy.
